# Cross-stress resistance in *Saccharomyces cerevisiae* yeast—new insight into an old phenomenon

**DOI:** 10.1007/s12192-016-0667-7

**Published:** 2016-01-29

**Authors:** Agata Święciło

**Affiliations:** Faculty of Agrobioengineering, Department of Environmental Microbiology, University of Life Sciences in Lublin, Leszczynskiego 7, 20-069 Lublin, Poland

**Keywords:** Environmental stress response, Acquired stress resistance, Same-stress resistance, Cross-stress resistance, *Saccharomyces cerevisiae* yeast

## Abstract

Acquired stress resistance is the result of mild stress causing the acquisition of resistance to severe stress of the same or a different type. The mechanism of “same-stress” resistance (resistance to a second, strong stress after mild primary stress of the same type) probably depends on the activation of defense and repair mechanisms specific for a particular type of stress, while cross-stress resistance (i.e., resistance to a second, strong stress after a different type of mild primary stress) is the effect of activation of both a specific and general stress response program, which in *Saccharomyces cerevisiae* yeast is known as the environmental stress response (ESR). Advancements in research techniques have made it possible to study the mechanism of cross-stress resistance at various levels of cellular organization: stress signal transduction pathways, regulation of gene expression, and transcription or translation processes. As a result of this type of research, views on the cross-stress protection mechanism have been reconsidered. It was originally thought that cross-stress resistance, irrespective of the nature of the two stresses, was determined by universal mechanisms, i.e., the same mechanisms within the general stress response. They are now believed to be more specific and strictly dependent on the features of the first stress.

## Stress response: an overview

Organisms in the natural environment are exposed to continuous changes in conditions, such as the availability of nutrients, osmotic concentration, temperature, or concentrations of cytotoxic substances. These changes, which are often unfavorable, necessitate an adequate response. Multicellular or motile organisms avoid the effects of suboptimal environmental conditions by changing their location or their physiological traits. Unicellular organisms lacking this capability possess a variety of mechanisms enabling them to quickly recognize and respond to a harmful factor, provided it does not occur in too great a quantity or concentration. One such mechanism is the stochastic process of gene rearrangement (stochastic switching), which causes certain cells in the population to become resistant to the effect of a harmful environmental factor. Another type of adaptation is adaptive resistance, appearing as a consequence of activation of the environmental stress response program.

Basic research on the stress response is conducted on a variety of organisms, among which *Saccharomyces cerevisiae* baker’s yeast has a well-established position. These are non-pathogenic, unicellular eukaryotic organisms present in the soil and water and on the surfaces of plants (leaves, fruits, and flowers). This yeast has also been associated throughout human history with the anthropogenic environment. In ancient times, it was used to produce beer and bread and is currently used in fermentation processes on an industrial scale (wine-making, beer brewing, distillation, and baking) and in the production of biomass (baker’s yeast, fodder yeast, yeast extracts, or single cell proteins (SCP)).

A variety of stress response mechanisms function in yeast, among which we distinguish an environmental stress response (ESR; triggered by many different stress factor) also known as the common environmental response (CER) and numerous mechanisms specific to particular stress factors (Causton et al. 2001; Gasch et al. 2000). The response to a specific environmental factor is unique, as it is consists of elements of both the specific and the environmental stress response. Moreover, elements of the stress response typical for bacterial cells have been identified (guanosine tetraphosphate (ppGpp) and guanosine pentaphosphate (pppGpp), which are mediators of the bacterial stringent response) in the mitochondria of yeast. This may additionally influence the phenotype of the yeast cell in stress conditions (Pao et al. 1977; Hamagishi et al. 1981). Studies by Yamada et al. (2003) and by Ochi et al. (2012)) confirmed that (p)ppGpp in yeast cells may regulate the expression of several genes, including stress responsive genes. Although activity of a ppGpp-synthesizing enzyme has not been observed in yeast, cytosolic synthesis of this unique nucleotide, owing to expression of the heterological gene Sj-RSH from the halophilic plant *Suaeda japonica* (homologous to the bacterial SpoT/RelA, encoding ppGpp synthetase) provided resistance to various types of stress, such as salt, osmotic stress, ethanol, hydrogen peroxide, high temperature, and freezing. Thus apart from stress response systems specific for eukaryotes, yeast cells may also have a latent conserved system analogous to the prokaryotic system in which ppGpp mediates.

The biological aim of the stress response is not only to protect cell components against the potentially dangerous effects of current stress factors but also to prepare them for a future harmful environmental factor of the same or a different type. The metabolic activity of the cell changes in response to a stress factor, due to the repression of synthesis of most proteins produced in the cell in normal physiological conditions and the induction of synthesis of a specific group of proteins known as stress proteins. These changes are accompanied by a temporary slowing down or inhibition of the division cycle.

Stress proteins include heat shock proteins (Hsp) and other proteins induced in a coordinated manner by various stress factors. These are mainly enzymes involved in cellular redox reactions and antioxidant defense, as well as enzymes active in the metabolism of carbohydrates and fatty acids, DNA repair, cell wall modification, metabolite transport, the functioning of mitochondria and vacuoles, autophagic processes, and intracellular signal transduction (Gasch et al. 2000).

The term Hsp was originally applied to a group of proteins induced selectively and in large quantities in heat shock conditions. It was later discovered that many heat shock proteins could also be strongly induced by other factors, such as viral infections, factors damaging DNA, amino acid analogues, or changes in environmental pH (Georgopoulos and Welch 1993). Some Hsp are produced constitutively, independently of environmental conditions. Their main functions are prevention of irreversible denaturation and restoration of native conformation (refolding) in denatured cellular proteins, newly synthesized proteins or those imported into cellular organelles, and proteolysis of aggregates of irreversibly denatured proteins. Chaperone proteins perform protective functions, while heat shock proteases and proteins associated with them are responsible for degradation of abnormally folded proteins. However, this seems to be merely a formal distinction, as many heat shock proteins exhibit both protective and proteolytic functions, depending on conditions (Padmanabhan et al. 2009).

The number of known factors inducing a response to stress (shock) is now so great that they are no longer catalogued. In general, they are classified as physical or chemical and as endogenous or exogenous. Exogenous factors are suboptimal environmental conditions of natural or anthropogenic origin. Anthropogenic stress factors include chemically synthesized products used on a large scale in modern agriculture, industry and households. The stress response can also be induced by starvation, which results when the supply of nutrients in the environment is depleted. In laboratory conditions, the most frequently applied types of stress are osmotic stress induced by NaCl, KCl, glycerol, or mannitol, heat stress induced by temperature changes (usually by raising the culture temperature, and less often by lowering it), oxidative stress induced by pro-oxidants such as hydrogen peroxide, menadione, or Paraquat, hyperoxia, and alcohol stress induced by the presence of ethyl alcohol.

Specific and general aspects of the stress response are distinguished, and depending on the cell’s reaction time from the moment the stress signal is received, we can distinguish early and late stress responses.

The specific response to stress is the result of activation of specific pathways for a particular type of stress. It includes activation of specific defense and repair mechanisms whose final effect is resistance to the same-stress factors. An example of the specific response is induction of synthesis of chaperones, which facilitate folding of other proteins, in conditions of heat shock. Folding reactions dependent on Hsp activity require the presence of ATP. A reduction in the ATP level usually results in increased expression of genes whose products are involved in energy acquisition processes (Hardie 1999). A mediator of the specific response to heat shock is the heat shock transcription factor (Hsf1p), a protein conserved from yeast to humans. The response to oxidative stress generated by menadione, hydrogen peroxide, or singlet oxygen involves strong activation of genes, mainly mediated by transcription factor Yap1p and/or Skn7p. The products of these genes are responsible for detoxification of hydrogen peroxide and superoxides (superoxide dismutase, glutathione peroxidase 3, and antioxidants specific for thiol groups). In these conditions, genes whose products are involved in redox reactions are activated as well (Brombacher et al. 2006; Stephen et al. 1995).

The specific response to increased external osmotic pressure includes activation of transcription factor Smp1p, mediated by protein kinases (MAPK) of the high osmolarity glycerol (HOG) pathway. It plays a significant role in regulation of the activity of a number of genes whose products are required for cells to adapt to osmotic stress conditions (De Nadal et al. 2003; Estruch and Carlson 1993). Depletion of glucose, the preferred source of carbon and energy in the living environment of yeast, leads to derepression of the synthesis of enzymes enabling the growth of these cells on alternative substrates such as ethanol or glycerol (Boy-Marcotte et al. 1998).

One form of the general stress response in baker’s yeast is the environmental stress response (ESR or CRS). This is a metabolic program associated with the activity of two homologous transcription factors, Msn2p and Msn4p (referred to as Msn2p/Msn4p), which are common to many different types of environmental stress. This program is characterized by inhibition of the expression of a large set of genes whose products determine cell growth, and activation of genes whose products control the processes of proteolysis and repair of damaged proteins, prevent oxidative damage, and take part in the reorganization of cellular structures, thereby maintaining metabolic homeostasis in the cell. Also activated in these conditions are genes whose products enable economic utilization of available carbon and energy sources or exploitation of alternative carbon sources (Chen et al. 2003; Gasch et al. 2000).

The early stage of the stress response includes a change in the activity of already existing enzymes and preparation of cells for the synthesis of new proteins, i.e., the perception of a stress stimulus and activation of stress signal transduction pathways and transcription factors regulating the activity of specific genes. At this stage, one of two different means of further transformations may be selected: slowing down growth in conditions of mild stress, or halting growth, but with a chance of survival, in conditions of severe stress.

The late stress response depends on the nature and intensity of the stress-inducing factor, and results in resistance of the cells to subsequent severe stress of the same type (same-stress resistance) or cross-stress resistance between pairs of different stresses. It includes biosynthesis of a specific set of proteins, posttranslational modifications of these proteins, and their specific reactions in the cell.

A stress-inducing factor can act in varying degrees of intensity. Mild stress usually refers to a factor acting in a sublethal dose, which does not reduce the survival rate of the cells but only slows down their growth.

Most often, such a stimulus activates the stress response, which mobilizes cellular defense mechanisms. Severe stress refers to conditions that temporarily halt the cell cycle at the G1 phase (Johnston and Singer 1980). The effects of severe stress usually cause damage to cellular macromolecules and induce structural and functional changes of varying scope. The final symptoms of severe stress may be acceleration of the aging process or induction of apoptosis or necrosis in the cell (Galluzzi et al. 2011; Madeo et al. 1999; Wawryn et al. 1999). Hence, the same stimulus, depending on the dose, can induce opposite physiological effects.

## Physiological aspects of acquired stress resistance

The phenomenon of acquired stress resistance (sometimes called the “adaptive response”) is universal, observed not only in *S. cerevisiae* budding yeast but in other micro-organisms and multicellular organisms, such as flatworms, plants, and mammals, including humans (Davies et al. 1995; Durrant and Dong 2004; Hecker et al. 2007; Kensler et al. 2007; Mitchell et al. 2009; Scholz et al. 2005). Acquired stress resistance plays an important role in the survival of various species in their natural environment.

Acquired stress resistance involves the acquisition of resistance to a severe stress under the influence of prior incubation in conditions of mild stress of the same type (same-stress resistance) or another type (cross-stress resistance).

Brief incubation of yeast cells in the presence of 0.7 M NaCl leads to an increase in the number of surviving cells exposed to a higher concentration of this compound—1.4 M NaCl (Trollmo et al. 1988; Varela et al. 1992). A similar effect has been observed in cells subjected to harmfully high temperatures (45 °C) following previous mild elevation of the culture temperature from 28 to 38 °C (Coote et al. 1991; Davies et al. 1995). Pre-incubation of cells in the presence of non-lethal doses of ethyl alcohol also leads to the appearance of resistance to lethal doses of the same factor (Vriesekoop and Pamment 2005). In these situations, the primary stresses induced resistance to a stronger dose of the same stressors, which is referred to as same-stress protection. In contrast, cross-stress protection refers to two different stresses applied in a particular order.

Cells preincubated in conditions of mild osmotic or ethanol shock become resistant to severe heat shock (Plesset et al. 1982; Trollmo et al. 1988; Varela et al. 1992) and oxidative shock (Berry and Gasch 2008; Berry et al. 2011). The appearance of heat tolerance has also been observed following prior incubation of cells in the presence of alcohol, weak acids, hydrogen peroxide, and copper ions, as well as after mutagenesis disrupting the structure of the cell wall (Carmelo et al. 1998; Cheng and Piper 1994; Coote et al. 1991; de Nobel et al. 2000). Mild heat shock and oxidative shock generated by hydrogen peroxide induce resistance to severe menadione shock, as well as shock caused by freezing (−20 °C) and thawing of yeast cultures (Flattery-O’Brien et al. 1993; Park et al. 1997). Mild heat shock also induces resistance to salinity, low environmental pH, oxidative shock, and the potentially lethal and mutagenic effect of the antibiotic bleomycin (Guan et al. 2012; Keszenman et al. 2000; Lewis et al. 1995; Zakrzewska et al. 2011).

It should be emphasized that the literature data referring to the conditions in which cross-stress resistance arises are not unambiguous. Different research teams often present conflicting results, which makes interpretation of the phenomenon more difficult. Data obtained by Trollmo et al. (1988) and Varela et al. (1992) indicate that mild heat shock does not offer protection against severe osmotic stress, but Lewis et al. (1995) and Berry and Gasch (2008) found that it does. Similar conflicting data have been collected in the case of cross-stress resistance to menadione and hydrogen peroxide following pre-incubation in the presence of these factors. Jamieson (1992) and Fernandes et al. (2007) noted that pre-incubation in the presence of menadione induces resistance to hydrogen peroxide, but the reverse order of application of these stresses does not produce this effect. On the other hand, Flattery-O’Brien et al. (1993) observed the reverse relationship, i.e., the appearance of resistance to menadione following pre-incubation in the presence of peroxide, and a lack of resistance to hydrogen peroxide following pre-incubation in the presence of menadione. These conflicting results have been the cause of consternation among both the readers and the authors of these studies, especially since cross-stress resistance is assumed to be a universal phenomenon occurring even phylogenetically distant organisms. Attempts to explain these discrepancies have been unsuccessful. Lewis et al. (1995) tested whether they could result from differences in the genotype of the strains analyzed, but found that all strains, both laboratory and industrial, had identical responses to the stresses tested alone or in combination. Therefore the ambiguities can be explained only by experimentation.

Aside from these ambiguous results, it is assumed that cross-stress resistance is common, although not a universal phenomenon for all combinations of stresses. Previous exposure of cells to heat shock causes resistance to alcohol shock, but reversing the order of application of these factors does not produce this effect (Piper 1995). Pre-incubation in conditions of mild oxidative shock generated by hydrogen peroxide leads to resistance to organic acids and ethanol, but mild ethanol shock does not protect against severe oxidative shock or the presence of organic acids (Semchyshyn 2014). Similarly, cells preincubated in the presence of sublethal doses of menadione became resistant to high doses of hydrogen peroxide, but the reverse order of application of these stresses did not lead to the development of a defense mechanism (Fernandes et al. 2007; Jamieson 1992).

Data indicating that shocks induce cross-stress protection in yeast irrespective of the order in which they are applied have been gathered as well. Mild osmotic shock has been found to protect yeast cells against the harmful effects of severe oxidative shock generated by hydrogen peroxide and vice versa – oxidative shock protected against the harmful effects of osmotic shock (Gasch 2007). Similarly, mild heat shock protected yeast cells against severe osmotic shock and mild osmotic shock provided protection against severe heat shock (Lewis et al. 1995).

These differences in the level of resistance induced by mild shocks can be systematized according to criteria proposed by Mitchell et al. (2009). According to the authors, the phenomenon of cross-stress resistance occurring irrespective of the order in which the stresses are applied is called symmetric cross-stress resistance, and cross-stress resistance resulting from stresses applied only in a particular order is asymmetric cross-stress resistance.

In light of these data, consecutively applied pairs of stresses—heat stress and alcohol stress; oxidative stress generated by hydrogen peroxide and ethanol stress; and oxidative stress generated by menadione and by hydrogen peroxide—induce asymmetric cross-stress resistance in yeast, whereas the pairs osmotic stress/oxidative stress generated by hydrogen peroxide and osmotic stress/heat stress induce symmetric cross-resistance.

## The biochemical basis of cross-stress resistance

In analyzing the molecular basis of the phenomenon of stress resistance (tolerance), various aspects should be distinguished. The basal stress tolerance to a stress factor depends on the efficiency of cellular defense and repair systems. In *S. cerevisiae* yeast, sensitivity to stress depends in particular on the effectiveness of vacuolar degradation, vesicular transport associated with endosomes, and nuclear functions such as RNA synthesis (Dudley et al. 2005; Hillenmeyer et al. 2008; Parsons et al. 2006; Okada et al. 2014). It is not, however, dependent on mechanisms regulated by stress. Neither the addition of antibiotics inhibiting the expression of genes associated with the stress response at various stages nor a lack of activity of basic Msn2/4p transcription factors mediating activation of the general stress response changes the level of sensitivity of cells to a single severe stress such as heat stress, osmotic stress, or the presence of hydrogen peroxide (Berry and Gasch 2008). Thus, remodeling of genomic expression triggered by an acute stress treatment is not required to survive that treatment but is required to survive subsequent stress exposure.

The basal stress tolerance can be raised as a result of mutation or adaptation to stress conditions. Within the adaptive response, two phenomena are distinguished, as described above: same-stress and cross-stress resistance. It is generally accepted that adaptive mechanisms, both resistant to the same type of stress and cross-stress resistance, are activated during the response to the primary mild stress and not the subsequent severe stress (Berry and Gasch 2008; Lewis et al. 2010). The mechanism of same-stress resistance probably depends on the activation of defense mechanisms specific for a particular type of stress; for example, resistance to repeated severe stress generated by hydrogen peroxide depends mainly on the level of activity of cytoplasmic catalase T. The characteristic high level of catalase T activity observed following mild stress generated by hydrogen peroxide, which is easily diffused through biological membranes, protects yeast cells against high concentrations of the compound by decomposing it into oxygen and water. Resistance to repeated exposure to menadione depends mainly on the level of reduced glutathione, which ensures a reductive environment in the cell, protecting sulfhydryl groups of proteins against oxidation and limiting lipid peroxidation (Fernandes et al. 2007). This mechanism is the main line of defense of cell structures against reactive oxygen species, which include the highly reactive superoxide anion radical generated by menadione.

The phenomenon of cross-stress protection is the effect of activation of both the specific and the general stress response by the same-stress factor (Berry and Gasch 2008; Zakrzewska et al. 2011). Application of cycloheximide (an antibiotic inhibiting protein translation) together with the primary mild stress has been shown to decrease resistance to a second stress, or to prevent acquisition of resistance to second stress. The role of Msn2/4p transcription factors, which mediate activation of the transcription of general stress response genes, was unclear in this process and was strongly dependent on the type of the primary mild stress (Berry and Gasch 2008).

Initially, when reasons for cross-stress resistance were sought at the biochemical level, it was assumed that they should be the same effectors of the general stress response that were induced in conditions of various mild shocks. The first candidates for the role of this type of factor were Hsp104p heat shock proteins and the disaccharide trehalose.

The protein Hsp104p (like the bacterial proteins ClpB, Sec18p, and Cdc48p) is one of the ATPases (associated with a variety of cellular activities) of the AAA family, which form a hexameric ring whose central channel has a diameter of 15 Ǻ. The role of Hsp104p involves disassembling protein aggregates generated in stress conditions via translocation of their central channel. In this process, Hsp104p works together with other heat shock proteins of the class Hsp70. Dissociation of protein aggregates and their refolding require substantial energy expenditures, as up to 12 ATP molecules are used for one cycle, and complete dissociation of the protein requires about 100 cycles (Doyle and Wickner 2009).

In *S. cerevisiae* yeast, Hsp104p is strongly induced during the specific response to heat stress, as well as in response to ethanol stress and in the presence of metals such as arsenic (Sanchez et al. 1992). It is one of the few heat shock proteins determining heat tolerance, as cells of the mutant *hsp104Δ* are extremely sensitive to heat shock (Sanchez and Lindquist 1990). It also plays a significant role in defense mechanisms against short-term, but not long-term desiccation. Hsp4p activity is thought to eliminate the harmful effects of this stress resulting from the aggregation of cytoplasmic and membrane proteins. The activity of Hsp 104p in these conditions can compensate for loss of trehalose (Tapia and Koshland 2014).

Trehalose is a non-reducing disaccharide found in many phylogenetically distant organisms, including plants, insects and invertebrates, and in both eukaryotic and prokaryotic microorganisms. The concentration of this sugar in cells is strongly dependent on environmental conditions (Richards et al. 2002). In *S. cerevisiae* yeast, trehalose synthesis is induced in conditions of ethanol, heat, oxidative shock, and dehydration and in the stationary phase of growth (Bleoanca et al. 2013; Gadd et al. 1987; Mahmud et al. 2009; François and Parrou 2001). This compound has documented activity protecting biological membranes, involving stabilization of polar groups of phospholipids and scavenging of free radicals, which protects the unsaturated fatty acids of membranes against peroxidation (Crowe et al. 1984; Herdeiro et al. 2006). Trehalose also functions as a chemical chaperone stabilizing both native and unfolded proteins, protecting them against aggregation (Tapia and Koshland 2014). The function of a reservoir of energy necessary for renaturation of damaged proteins is also attributed to trehalose (Singer and Lindquist 1998).

Trehalose and Hsp104p have been shown to exhibit a synergistic effect in the case of resistance to high temperature in yeast cells in the stationary phase of growth (Elliott et al. 1996). Trehalose can influence the renaturing activity of Hsp104p via two independent mechanisms. It conditions full activity of transcription factor Hsf1p, which activates the transcription of numerous genes, including *HSP104*, in heat shock conditions (Conlin and Nelson 2007). The high level of activity of this factor probably results from the ability of trehalose, observed by various authors, to stabilize the tertiary structure of the carboxyl-terminal of the protein’s activation domain (Bulman and Nelson 2005; Conlin and Nelson 2007). The ability of trehalose to stabilize unfolded cellular proteins can also contribute to Hsp104p-mediated inhibition of their renaturation (Singer and Lindquist 1998). This explains the dynamics of changes in trehalose concentration during the stress response, e.g., its rapid degradation directly after glucose is added to a yeast culture to initiate the fermentation process (Wera et al. 1999).

Support for the hypothesis concerning the key role of Hsp104p and trehalose in mechanisms of cross-stress resistance can be found in results indicating that accumulation of trehalose and biosynthesis of Hsp104p depend on the activity of Msn2/4p transcription factors. In the promoter of *HSP104* and of genes encoding subunits of the trehalose synthase complex, a stress responsive element (STRE) sequence has been discovered which is recognized by these transcription factors (Treger et al. 1998; Winderickx et al. 1996). It should be emphasized, however, that not all genes for the various subunits of trehalose synthase have the same pattern of expression in the same-stress conditions. In addition, on the promoters of both HSP104 and TPS1, (which encodes trehalose-6-phosphate synthase), there is an additional regulatory sequence in the form of an heat shock element (HSE), which is recognized by transcription factor Hsf1p (Grably et al. 2002). However, according to the latest research the Hsf1-dependent transcription activity of TPS1 gene has no role in thermotolerance mechanisms (Petitjean et al. 2015).

The role attributed to trehalose as a protectant against various types of stress was the result of various observations, such as its ability to stabilize the structures of various molecules in *in vitro* studies; a correlation between trehalose accumulation under the influence of stresses and the level of resistance of such cells to stresses; or hypersensitivity to stresses in mutants incapable of biosynthesis of trehalose. These last two experimental approaches made it impossible to differentiate effects induced by trehalose itself from those with less obvious causes, such as metabolic flux, the independent function of biosynthetic enzymes, or intermediates in trehalose biosynthesis.

The latest literature reports have led to a revision of views on the role of trehalose in mechanisms against stress in yeast. Experiments by Gibney et al. (2015) and Tapia and Koshland (2014)) on yeast mutants, in which the concentration of intracellular trehalose was manipulated, clarified the role of trehalose in resistance to different stresses. The experiments clearly show that only in the case of long-term dehydration/desiccation (weeks to months) is trehalose a significant and sufficient factor determining resistance to this type of stress (Tapia and Koshland 2014). This trehalose-induced tolerance is independent of utilization of trehalose as an energy source, de novo synthesis of other stress effectors, or the metabolic effects of trehalose biosynthetic intermediates, indicating that a chemical property of trehalose is directly responsible for desiccation tolerance (Tapia et al. 2015).

On the other hand, the specific role previously attributed to trehalose in protection against severe heat stress was not confirmed in experiments by Gibney et al. (2015) A high intracellular level of trehalose obtained by loading the cells with trehalose imported from the external environment did not fully protect the cells of a wild-type strain or a *tps1* mutant (incapable of synthesizing trehalose-6 phosphate from glucose-6 phosphate and the glycosyl unit from UDP-glucose) against the effects of severe heat stress. It also had no effect on the survival of these cells in conditions of severe heat stress following prior adaptation to severe stress by pre-incubation in conditions of mild heat stress.

Trehalose loading of cells of a tps2∆ mutant incapable of synthesizing trehalose from trehalose-6 phosphate led to improvement in its survival rate in conditions of severe heat stress, both with and without pre-incubation in conditions of mild heat stress. Interestingly, cells of the wild-type strain loaded with trehalose showed a similarly high survival rate in these conditions as the delta tps2∆ cells. However, this effect was not trehalose specific, as similar effects were induced by loading the wild-type cells with maltose, another disaccharide.

There is evidence that the protection against severe heat stress observed in the presence of trehalose in yeast cells of both the wild-type strain and the mutant incapable of trehalose synthesis was due to the effect of trehalose on the cell’s metabolism (which means that this is an indirect effect of trehalose activity). Intracellular accumulation of trehalose inhibits the growth of yeast cells and at the same time leads to changes in the gene expression profile, similar to changes characteristic of the environmental stress response program. The magnitude of the changes is correlated with the level of growth inhibition (Gibney et al. 2015). Thus resistance to strong heat shock may in this case be the result of activation by trehalose of the ESR program and production within this program of other thermoprotective effectors. In this case, the effect of an intermediate in the trehalose biosynthesis pathway, trehalose-6 phosphate, a compound with documented activity as a metabolic regulator, may also play a role (Hohmann et al. 1996). This hypothesis is supported by the fact that cells of the *tps2* mutant have a high concentration of trehalose-6 phosphate because they lack the activity of the enzyme that utilizes it to produce trehalose.

The role of trehalose-6 phosphate in defense mechanisms against stresses was also confirmed in a study by Petitjean et al. (2015). These authors used a strategy analogous to the one described above, raising the level of intracellular trehalose from external sources, and inactivated intracellular biosynthesis of this disaccharide. They found that activity of Tps1 (trehalose 6-phosphate synthetase enzyme) and not that of trehalose itself is the key to survival in conditions of heat stress, oxidative stress or desiccation. The mechanism by which Tps1 determines resistance to heat shock involves maintaining a suitable level of ATP. These results confirm that trehalose-6 phosphate is not only an intermediate in the trehalose biosynthesis pathway but also a molecule with pleiotropic activity, including resistance to stresses.

In light of these data, the role of Hsp 104 and trehalose in resistance to various stresses appears to have been overestimated in earlier literature reports, which were based mainly on indirect evidence. Additionally, both of these substances have been shown to rapidly undergo degradation immediately after application of a stressor. This ruled it out as one of the possible universal factors determining cross-stress protection, because it was assumed that the factor playing the key role in cross-stress protection is synthesized during the primary stress and must be present in the cells during the second, severe stress. Therefore, further research has focused on the search for long-lived proteins that would serve as a specific proteomic memory of the primary stress (Guan et al. 2012). It was initially suggested that these could be enzymatic proteins taking part in the synthesis of cellular protectants, such as trehalose, glycerol or chaperones, but the literature data have not confirmed this. These criteria have, however, been met by cytoplasmic catalase T, an enzymatic protein present in yeast cells that have been subjected to osmotic stress (Berry et al. 2011).

The number of transcripts of the gene *CTT1* encoding catalase T is very low prior to stress but has been shown to increase sharply just a few minutes after the application of mild osmotic stress (Lipson et al. 2009). The maximum level of transcripts of this gene is noted 30 min after stress is applied, but within the next 30 min, it returns to its pre-stress level. Therefore, if mild osmotic stress lasts for one hour, then after this time no change is observed in the level of *CTT1* transcripts in comparison with the pre-stress level, but there are perceptible changes in the level of catalase, which at this time displays its maximum level of activity (Guan et al. 2012; Święciło et al. 2000). The high level of catalase activity persists for the next half hour and then slowly decreases, though for the next 330 min, it is still higher than in unstressed cells. If we assume that the average generation time for yeast cells of a wild-type strain is 80 min, then catalase is transferred to at least four daughter cells (Święciło et al. 2000). During this entire period (about 6 h after the stress factor is removed from the living environment of the yeast), the mother cells and the daughter cells, which were never subjected to osmotic stress, were resistant to high doses of hydrogen peroxide (Guan et al. 2012).

Direct evidence of the decisive role of catalase T in resistance to hydrogen peroxide following osmotic stress was provided by an experiment in which catalase synthesis was induced in non-stress conditions. The study used a yeast strain in which the *CTT1* gene was replaced by *Flag-CTT1* introduced on the plasmid, whose promoter was susceptible to regulation by exogenous estradiol (Gao and Pinkham 2000). Yeast cells stimulated with estradiol exhibited a high level of catalase activity and a high level of resistance to hydrogen peroxide, persisting for over 360 min after the estradiol was removed from the environment. In contrast, isogenic cells of the strain *ctt1*Δ in the same experimental conditions showed no increase in either catalase activity or resistance to hydrogen peroxide (Guan et al. 2012).

Yeast catalase T, however, does not appear to be a universal factor determining resistance to hydrogen peroxide. No increase in catalase T activity was noted following mild menadione shock, and cells of the *ctt1Δ* strain, despite a complete lack of cytoplasmic catalase T activity, were still capable of acquiring resistance to hydrogen peroxide (Fernandes et al. 2007). Following mild heat shock and after exposure to dithiothreitol (DTT), while transcription of *CTT1* was stimulated (to an unequal degree), in both cases only a small amount of catalase was accumulated.

Resistance to hydrogen peroxide following mild menadione stress, heat stress or stress generated by DTT depended on the concentration of reduced glutathione, which is the main component of the cellular redox buffer (Berry et al. 2011; Fernandes et al. 2007). A lack of activity of glutathione synthetase (Gsh1p), the first enzyme of the glutathione synthesis pathway, and glutathione reductase (Grl1p), an enzyme synthesizing reduced glutathione at the expense of NADPH, prevented acquisition of resistance to hydrogen peroxide. However, these proteins were not induced by the mild stresses applied (Berry et al. 2011). In the presence of DTT, an approximately 2.5-fold increase was observed in the activity of glutathione peroxidase (Gpx2p), an alternative enzyme to catalase taking part in the decomposition of hydrogen peroxide. However, after heat and osmotic shock the increase in the activity of this enzyme was marginal (1.3 times). The concentration of the isoenzyme Gpx1p could not be measured in the same experimental conditions, but the number of transcripts of the gene *GPX1* encoding this protein increased following mild heat and osmotic shock (Berry et al. 2011). Deletion of either *GPX1* or *GPX2* did not lead to defects in acquired tolerance, probably due to their complementary cellular functions (Avery and Avery 2001).

The data presented show that in cross-stress resistance mechanisms we can distinguish elements that are necessary but not sufficient for the development of cross-stress resistance (e.g., synthetase and glutathione reductase) and sufficient elements, including components induced by stress factors, such as catalase T or glutathione peroxidase. The genes required for the acquisition of tolerance to severe stress are expressed constitutively and determine the basal stress tolerance. Interestingly, in the case of stress generated by DTT, most genes sufficient for activation of cross-stress resistance have been found to be regulated negatively, i.e., their transcription was inhibited in response to the primary mild shock (Berry et al. 2011).

A great deal of new information concerning necessary and sufficient elements for cross-stress resistance has been provided by studies on deletion mutants (Berry et al. 2011; Zakrzewska et al. 2011). According to Berry et al. (2011), among 841 mutant strains only 28 were identified that were incapable of acquiring resistance to hydrogen peroxide following prior incubation in conditions of mild heat stress or osmotic stress or after exposure to DTT. This means that in the case of these three mild stresses only the expression of 28 genes (involved in basic metabolic processes) determines resistance to hydrogen peroxide. These account for only 5.6 % of all genes activated by DTT, 9.1 % of all genes activated by mild heat shock and 12.4 % of all genes activated by mild osmotic shock. Interestingly, these overlap only slightly with the set of genes induced by mild oxidative shock that are necessary to activate resistance (Kelley and Ideker 2009).

The role of these 28 genes in acquired resistance to hydrogen peroxide was varied and strongly dependent on the type of initial mild stress. They were divided into functional categories depending on the type of cellular process they take part in. Only genes encoding proteins involved in DNA repair or vacuolar processes and proteins that are negative regulators of the Ras (IRA1) and TOR (NPR2, NPR3) signaling cascade were equally necessary for acquisition of resistance to hydrogen peroxide irrespective of the type of initial stress.

The significance of the remaining genes in this process varied depending on the specific character of the primary mild stress. After mild osmotic stress, a more important role was played by genes involved in proteolysis and the HOG signaling pathway, which activates mechanisms protecting against severe osmotic stress. After mild heat stress, genes involved in DNA repair, protein transport and late transport from endosomes to vacuoles played an important role in this process, while following reductive stress generated by DTT, the important genes were mainly those involved in ubiquitin-dependent and ubiquitin-independent protein degradation, ribosome functions and regulation of translation. The data presented show that different sets of genes and different cellular processes controlled by them can take part in mechanisms of cross-stress resistance to the same factor.

Zakrzewska et al. (2011) used a similar experimental approach involving analysis of 4066 deletion mutants to identify functional categories of genes necessary for acquisition of cross-stress resistance to severe oxidative stress, heat stress, and low pH (generated by the presence of a weak organic acid) following mild heat shock. As in the case of the research described above, these were genes determining basic cellular functions such as vesicular transport, reorganization of the cytoskeleton, transcription, mRNA modification, translation, and import and export of regulatory factors from the nucleus.

In addition, this study confirmed an inverse relationship between cell growth rate and the level of acquired stress resistance (Lu et al. 2009; Zakrzewska et al. 2011), i.e., the slower the cells grew in stress conditions, the more resistant they were. This was observed not only in the case of deletion mutants but also in wild-type cells growing in growth-restricting conditions (chemostatic growth with low glucose supply). Slow growth, irrespective of its cause (mutation or glucose starvation) led to an increase in resistance to severe heat stress, low pH, and oxidative stress generated by hydrogen peroxide. The authors suggest that a decreased growth rate or complete cessation of growth can be both the final effect and the cause of stress. The mechanism leading to slowed growth is an integral part of the stress response program (Regenberg et al. 2006). This claim is supported by observations that suppression of mutations leading to growth defects in slow-growing but stress-resistant mutants led to decreased tolerance for severe stresses. Both processes (yeast cell growth and adaptive resistance to severe stress) were found to be controlled by a set of genes whose products are involved in basic metabolic processes, such as ribosome biogenesis, transcription catalyzed by polimerase II, and mitochondrial functions. These are mainly genes characteristic of the general environmental stress response (ESR) program (Gasch et al. 2000). Genes controlling growth processes belong to a large set (about 600 genes) whose transcription is inhibited in the stress response. Weakening of the expression of a large set of genes whose products take part in protein biosynthesis and growth appears to be purposeful, given the benefits arising from limiting energy expenditures on biochemical transformations which are of secondary importance in stress conditions. This hypothesis is consistent with observations that mutants lacking certain genes from this group have a higher growth level in stress conditions than cells of the wild-type strain (Giaever et al. 2002; Hillenmeyer et al. 2008; Parsons et al. 2006).

A significant factor determining adaptive tolerance for heat stress and low pH (but not oxidative stress) is the activity of the Rpd3 histone deacetylase complex, which regulates gene transcriptions both positively and negatively in response to stress (Alejandro-Osorio et al. 2009; Zakrzewska et al. 2011). Earlier studies demonstrated the importance of this complex in acquired resistance to osmotic stress and multidrug resistance (De Nadal et al. 2004). The data presented indicate that a functional role in adaptive stress resistance is played by the products both of genes whose transcription is activated (including genes regulated by Msn2/4p) and those whose transcription is inhibited in the general stress response.

## The genetic basis of cross-stress resistance

As discussed above, cross-stress resistance is a phenomenon resulting from the presence in the cell of specific metabolites generated by the effect of mild shock. Their quantity and quality depend on the nature and intensity of the stress stimulus, but their qualitative composition often exceeds the current need for protective substances. This functional “excess” of metabolites generated in conditions of mild stress may ensure protection against severe stress of another type. This hypothesis formed the basis for the first model explaining the mechanism of cross-stress resistance. It was proposed by Martınez-Pastor et al. (1996) and by Schmitt and McEntee (1996) and explained the “excess” of the same metabolites occurring in conditions of different types of stress as the effect of the activity of the Msn2/4p transcription factors, referred to as the main factors of the environmental stress response. Genes whose transcription was activated via these factors were called general stress response genes (Causton et al. 2001; Gasch et al. 2000).

**Fig. 1:**
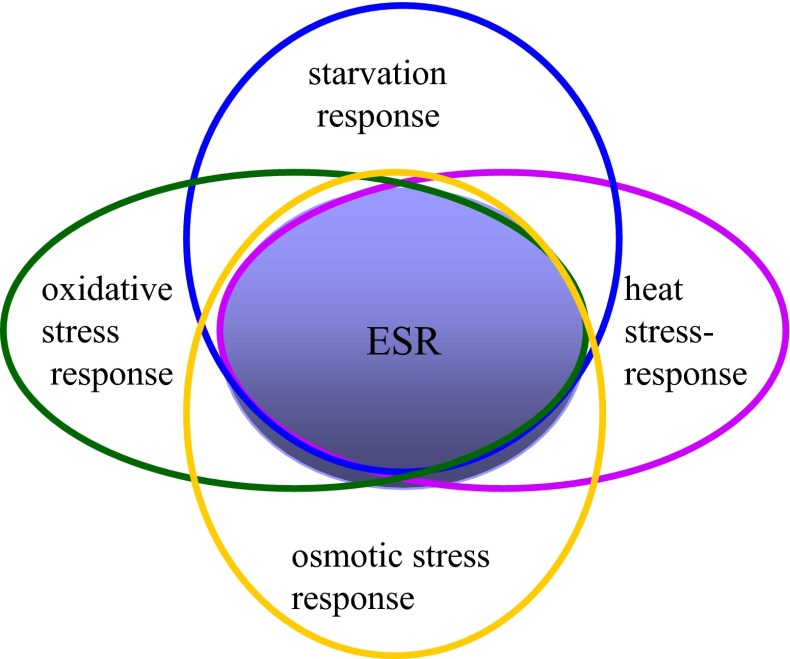
Environmental stress response as the basis of cross-stress resistance

Figure 1 presents the first model explaining the phenomenon of cross-stress resistance as a consequence of activation of the response to environmental stress by means of activation of genes of the environmental stress response. Because the environmental stress response is activated in conditions of different stresses, the metabolic picture of the response to them, and thus their physiological effects, overlap to some extent. This overlap may explain the phenomenon of cross-stress resistance.

The ESR involves about 900 genes in *Saccharomyces cerevisiae* yeast, which is 14 % of all yeast genes (Ball et al. 2001). Transcription of about 600 genes is inhibited in the ESR, while about 300 genes are activated (Ball et al. 2001; Causton et al. 2001; Gasch 2007). Of these, about 50 genes are activated by the Msn2/4p transcription factors (Gasch et al. 2000). These are genes encoding certain antioxidant enzymes, proteases, chaperones, housekeeping enzymes, and other proteins involved in repair processes or in the removal of damaged biomolecules and restoration of metabolic homeostasis. It was expected that this group of genes would include those whose products determine cross-stress resistance, and in fact a number of metabolites were identified as being responsible for resistance in specific conditions. Nevertheless, numerous doubts arose as to the premises of this model.

Most products of general stress response genes are not essential for adaptive resistance to severe stress to appear, as the deletion of single genes coding for these proteins does not affect (or affects only slightly) the level of sensitivity of cells to stress factors (Estruch and Carlson 1993; Martınez-Pastor et al. 1996).

Furthermore, the effects of depriving cells of Msn2/4p transcription factors are unclear. Earlier literature data indicate that deletion of the genes *MSN2* and *MSN4*, which encode these factors, leads to hypersensitivity to several different types of stress, such as heat, osmotic, and oxidative stresses or stress caused by carbon source depletion in the environment (Martınez-Pastor et al. 1996). The results of later studies, however, suggest that this type of mutation does not change basal stress tolerance (Berry and Gasch 2008; Zakrzewska et al. 2011). According to Zakrzewska et al. (2011), *msn2Δmsn4Δ* mutations also do not affect the ability to acquire resistance to severe oxidative or heat stress or to low pH following mild heat stress. A study by Berry and Gasch (2008) revealed that the effects of this type of mutation are strongly dependent on the type of the first stress. Deletion of both genes prevents acquisition of resistance to osmotic stress and stress induced by hydrogen peroxide following prior incubation in conditions of heat and osmotic shock. On the other hand, it has only a slight effect on resistance to hydrogen peroxide and osmotic stress after pre-incubation in conditions of mild oxidative stress. This study also showed that the roles of Msn2p and Msn4p in the cross-stress resistance mechanism were not entirely redundant.

It is additionally postulated that apart from these long-known factors in the general stress response, there are other mechanisms mediating activation of cross-stress resistance. This has been demonstrated in studies on the *S. cerevisiae* mutant *fil1* (fermentation-induced loss of stress resistance) with reduced adenylate cyclase activity. This mutation is expressed as a high level of resistance to stress irrespective of the availability of glucose, the preferred source of carbon and energy (Van Dijck et al. 2000). A reduction in the amount of glucose in the growth environment is a stress factor in yeast that activates the general stress response mechanism via the cAMP/PKA pathway.

Deletion of the single gene *TPS1*, *HSP104*, or both of the genes *MSN2* and *MSN4* in cells of a wild-type strain substantially decreased resistance of these cells to heat shock, but this type of mutation only very slightly changed the resistance of the *fil1* mutant. Similar results were observed following removal of all four genes at the same time. Cells of the quadruple mutant *tps1*Δ*hsp104*Δ*msn2*Δ*msn4*Δ were extremely sensitive not only to heat stress but also to salt stress, osmotic stress and oxidative stress. However, an additional fifth *fil1* mutation in this strain largely eliminated the detrimental effects caused by the lack of a general stress response program, increasing resistance to the stresses analyzed (Versele et al. 2004). Hence, the level of cross-stress resistance is dependent not only on high concentrations of trehalose and Hsp104p and overexpression of general stress response genes mediated by Msn2p and Msn4p, but on other factors as well. For example, it has been suggested that posttranslational modifications of proteins or the interaction of various transduction pathways of environmental signals may play this role.

Another model based on the same premises postulated that transcription factors other than Msn2/4p may also be involved in the activation of general stress response genes.

Transcription factors in eukaryotes are the main mechanism controlling gene expression. They regulate the rate at which active transcription complexes with specific affinity for particular promoter sequences are formed. Knowledge of transcription factors involved in the stress response was significantly enhanced when the results of genetic experiments (dynamic profiles of gene expression and a list of promoter sequences interacting with transcription factors) were combined with statistical analysis techniques. The number of transcription factors taking part in the response to different stresses, both those confirmed experimentally and those predicted using various statistical models, is substantial. When the same transcription factors mediate activation of the response to different stresses, they are classified as transcription factors of the general stress response. Their activity, coordinated with other specific factors, leads to a characteristic overlapping of their effects. Overlapping of the effects of the stress response at the regulator level is also sometimes referred to as regulatory cross-talk (Wu and Chen 2009).

The program Stress Transcription Factor Identification Algorithm (STFIA) was used to predict the role of 47 transcription factors in the response to the most frequently studied environmental stresses (heat, osmotic, oxidative generated by menadione or hydrogen peroxide, nitrogen starvation, and amino acid starvation) (Wu and Chen 2009). Factors with a confirmed role in the response to these stresses accounted for 51 % of all those identified in the program, while factors with partially confirmed regulatory functions constituted 32 %. New factors, with no confirmation of their regulatory roles in the literature, accounted for 17 % (eight proteins).

**Fig. 2:**
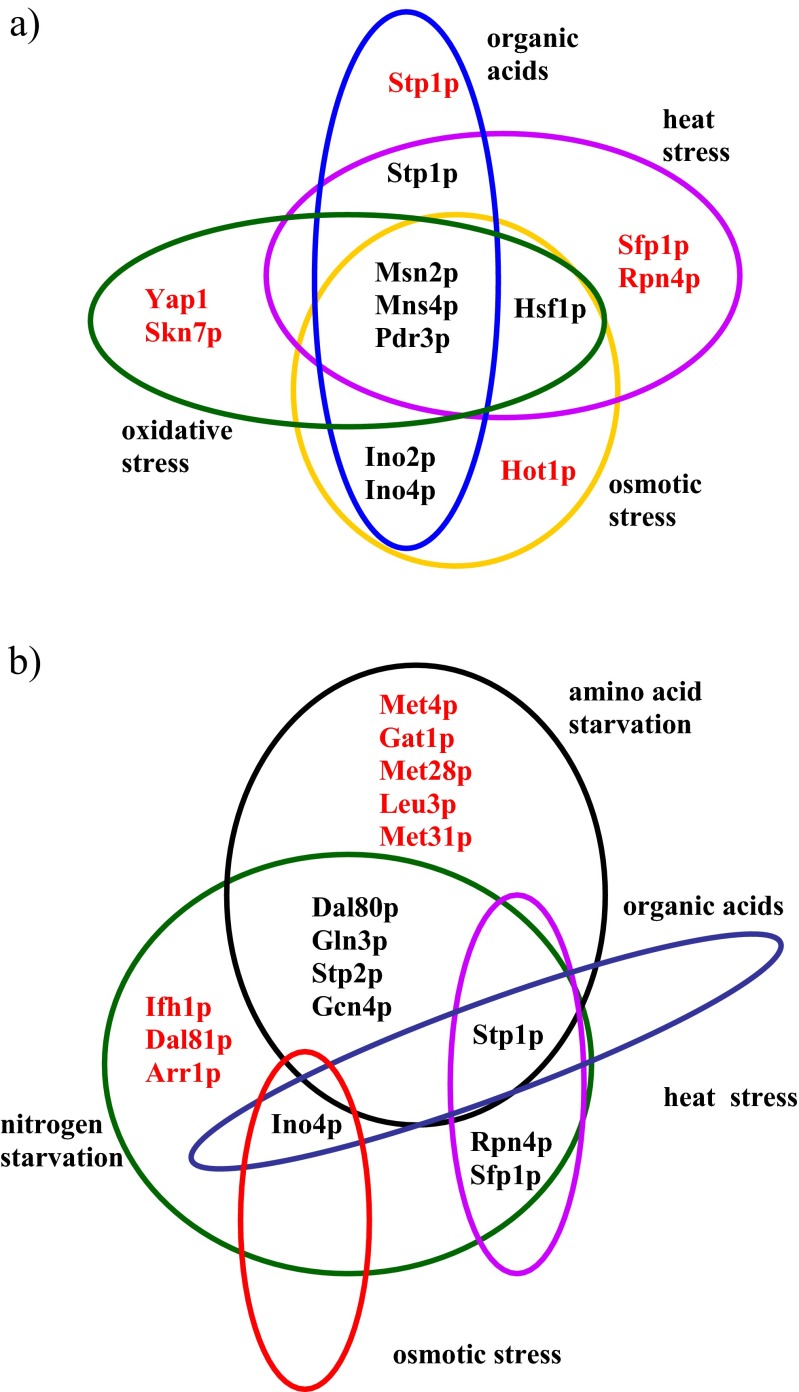
Transcription factors specific for a particular type of stress or common transcription factors mediating the response to **a** heat, oxidative, and osmotic stresses and the presence of organic acids, **b** nitrogen and amino acid starvation, and heat and osmotic shock (modified from (Wu and Chen 2009)

Known transcription factors (TF) identified in the response to heat stress were Msn2p, Msn4p, Hsf1p and Pdr3p, and the role of others, Stp1p, Sfp1p, and Rpn4p, was predicted. In the response to oxidative stress, in addition to well-characterized TF, i.e., Msn2p, Msn4p, Yap1p, and Skn7p, new ones were identified as well, i.e., Pdr3p and Hsf1p. In the osmotic stress response factors known from previous research, i.e., Hot1p, Msn2p, and Msn4p, were identified, and the role of Pdr3p, Hsf1p, Ino2p, and Ino4p was suggested. In the response to stress induced by the presence of weak organic acids in the environment, the known factors Msn2p and Msn4p were identified, and the role of Pdr3p, Ino2p, Ino4p, and Stp1p was suggested. The participation of known TF Gln3p, Dal80p, Dal81p and Gcn4p was observed in activation of the response to nitrogen starvation, and the activity of others was predicted: Stp1p, Stp2p, Sfp1p, Arr1p, Rph4p, Ifh1p, and Ino4p. In the response to amino acid starvation (generated by a deficiency of amino acids in the growth environment) the activity of known TF Gcn 4p, Dal 80p, Met 28p, Gln 3p, Met 4p, Leu 3p, Gat1p, and Met31p was confirmed, and that of Stp1p and Stp2p was predicted. In the case of heat stress, osmotic stress and stress induced by the presence of organic acids, regulatory cross-talk was noted for Msn2p, Msn4p, and Pdr3p, while in the case of stress induced by nitrogen and amino acid starvation, it was observed in Gcn4p, Gln3p, Dal80p, Stp1p, and Stp2p. Figure 2 shows these relationships in graphic form.

A methodologically similar approach was used to create a different model of interactions between stress and transcription factors (Chen et al. 2009). In this model, the set of transcription factors taking part in the response to a specific stress stimulus is defined as a network of transcriptional sensors, and as in the previous solutions, the involvement of the same factors in the response to different stresses is treated as regulatory cross-talk. This model postulates that as many as 64 transcription factors are involved in the response to oxidative stress induced by menadione, 58 transcription factors mediate the response to diamides, 55 transcription factors are active in the response to heat shock, 44 in the response to cold shock, 58 in the response to hypertonic conditions, and 57 in the case of hypotonic conditions. According to this model, 85 transcription factors are responsible for regulating gene activity in conditions of at least three environmental stresses. Among these factors, known activators of the general stress response, Msn2p and Msn4p, were identified. Segal’s research team (Segal et al. 2003) predicted 21 factors in this group. The same five transcription factors (Bas1p, Fkh1p, Gcr1p, Phd1p, and Swi6p) probably control the response to at least six environmental stresses, and two of these, Bas1p and Fkh1p, were previously predicted by Segal et al. (2003). Only Gcr2p, which activates yeast genes controlling the glycolysis pathway, probably mediates the response to all of the stresses analyzed (except exposure to DTT) (Uemura and Jigami 1992).

The relationships presented explain the mechanism of cross-stress resistance as an expression of the overlap of regulators, i.e., their cross-talk in different stress conditions. However, on the basis of this model, it is difficult to explain the asymmetric cross-stress resistance often observed in yeast.

Another model, based on observations by Berry and Gasch (2008) and Berry et al. (2011), suggests that the mechanism of cross-stress resistance is not dependent on a universal mechanism including the activity of the same transcription factor or factors and/or activation of the same set of genes but is the result of activation of a specific stress response program induced by the first mild stress. This line of reasoning is confirmed by observations that acquisition of resistance to hydrogen peroxide resulting from pre-incubation in various stress conditions takes place via activation of a different group of genes in each case (Berry et al. 2011; Kelley and Ideker 2009). The final effects of activation of different sets of genes may be the same, i.e., they may lead to resistance to different types of severe stress, because cells have numerous alternative and/or low-specificity defense and repair mechanisms.

## Conclusions

Cross-stress resistance is a universal phenomenon observed in prokaryotic and eukaryotic microorganisms as well as in multicellular organisms. Research on the nature of this phenomenon has continued uninterrupted for over 40 years. As research techniques have advanced, views on the molecular basis and biological meaning of this phenomenon have changed systematically. In the 1980s and 1990s, same-stress resistance and cross-stress resistance were already being treated as an inherent attribute of the response of *S. cerevisiae* yeast to various types of environmental stress. These two phenomena, arising as a consequence of activation of the response to a mild stressor, ensure protection against a severe stressor of the same or a different type, respectively. In this regard, they can be treated as the result of the overlap of the effects of two of the same or different stressors, differing in severity. Hence, the causes of the two phenomena have been sought in common features of the stress response. In the initial stage of research, the general ESR in *S. cerevisiae* yeast was commonly accepted as the basis of cross-stress resistance. This reasoning was supported by observations that the program was switched on by different environmental signals via different signaling pathways, which coincided at the level of the Msn2/4p transcription factors, known as activators of general stress response genes. Moreover, many products of these genes were thought to be the main determinants of resistance to severe stress and of cross-stress resistance. Thus, it was initially supposed that the universal mechanism of the general stress response underlies the phenomenon of cross-stress resistance.

Currently, there is a great deal of data indicating that in addition to elements of the general stress response, mechanisms specific for the conditions of the first mild stress also play a role in cross-stress resistance. Depending on the specific character of the first stress, cross-stress resistance mechanisms are dependent on the products of different genes, both induced and repressed. Coordinated regulation of both sets of genes explains the connection between the rate of cell growth and the level of resistance to different severe environmental stresses. Specific regulation of gene activity depending on the type of mild stress is consistent with currently postulated models suggesting the involvement of numerous (known and predicted) transcription factors mediating activation of the response to different stresses. In this context, the phenomenon of cross-stress resistance is explained as the effect of the involvement of the same transcription factors in the response to different stresses, which has been termed “regulatory cross-talk” in the literature. Cross-stress resistance may also result from a low degree of specificity of defense and/or repair mechanisms or from the existence in cells of alternative mechanisms leading to the same biochemical effects. Cellular defense mechanisms, usually organized on many levels, both in terms of subcellular localization and the specific character of the processes taking place, and composed of both stress-induced and constitutively synthesized elements, ensure preservation of the integrity of cell structure and functions in conditions of different types of severe stress.
